# Comprehensive data optimization and risk prediction framework: machine learning methods for inflammatory bowel disease prediction based on the human gut microbiome data

**DOI:** 10.3389/fmicb.2024.1483084

**Published:** 2024-10-01

**Authors:** Yan Peng, Yue Liu, Yifei Liu, Jie Wang

**Affiliations:** ^1^School of Management, Capital Normal University, Beijing, China; ^2^School of Mathematical Sciences, Capital Normal University, Beijing, China

**Keywords:** gut microbiome, inflammatory bowel disease, novel risk warning framework, machine learning, data imputation, parameter optimization

## Abstract

Over the past decade, the prevalence of inflammatory bowel disease (IBD) has significantly increased, making early detection crucial for improving patient survival rates. Medical research suggests that changes in the human gut microbiome are closely linked to IBD onset, playing a critical role in its prediction. However, the current gut microbiome data often exhibit missing values and high dimensionality, posing challenges to the accuracy of predictive algorithms. To address these issues, we proposed the comprehensive data optimization and risk prediction framework (CDORPF), an ensemble learning framework designed to predict IBD risk based on the human gut microbiome, aiding early diagnosis. The framework comprised two main components: data optimization and risk prediction. The data optimization module first employed triple optimization imputation (TOI) to impute missing data while preserving the biological characteristics of the microbiome. It then utilized importance-weighted variational autoencoder (IWVAE) to reduce redundant information from the high-dimensional microbiome data. This process resulted in a complete, low-dimensional representation of the data, laying the foundation for improved algorithm efficiency and accuracy. In the risk prediction module, the optimized data was classified using a random forest (RF) model, and hyperparameters were globally optimized using improved aquila optimizer (IAO), which incorporated multiple strategies. Experimental results on IBD-related gut microbiome datasets showed that the proposed framework achieved classification accuracy, recall, and F1 scores exceeding 0.9, outperforming comparison models and serving as a valuable tool for predicting IBD onset risk.

## 1 Introduction

Inflammatory bowel disease (IBD), which includes ulcerative colitis (UC) and Crohn’s disease (CD), is a group of chronic inflammatory disorders of the gastrointestinal tract (Flynn and Eisenstein, 2019). IBD is associated with an increased risk of intestinal malignancies (Faye et al., 2022), and it can also lead to complications involving the joints, skin, eyes, and central nervous system (Rogler et al., 2021). Additionally, patients with IBD frequently experience comorbid depression and anxiety (Bisgaard et al., 2022). Although no specific pathogen has been definitively implicated in the etiology of IBD, a growing body of evidence suggests a significant association between the human gut microbiome and the development of IBD (Kostic et al., 2014).

The development of high-throughput sequencing technologies has enabled researchers to capture a comprehensive snapshot of the microbial community of interest (Almeida et al., 2019). Among these, 16S rRNA gene sequencing stands out as an efficient and cost-effective method for identifying and classifying bacteria and archaea within microbial populations (Johnson et al., 2019).

Although new technologies have significantly enhanced our ability to characterize the human gut microbiome and its potential in predicting IBD, several key challenges remain in effectively utilizing these data to construct predictive models. Firstly, due to the high-dimensional sparsity of microbiome data and the limitations of sequencing technologies, data missingness is a prevalent issue. Most current studies employ simple mean imputation, zero-filling methods or K-nearest neighbors (KNN) imputation (Liñares-Blanco et al., 2022), which fail to adequately capture the intrinsic structure and complex relationships within the data, potentially leading to decreased model performance. While multiple imputation by chained equations (MICE) is widely utilized for data imputation, it possesses several constraints. Its performance could be considerably influenced if the data fails to meet the assumption of missing completely at random (MCAR) (Azur et al., 2011). Furthermore, MICE is susceptible to the selection of model parameters (Doove et al., 2014), particularly with non-linear relationships or interactions, which might result in less reliable imputation outcomes. The approach also brings in uncertainty, as the results may vary across different datasets or subsets of the same dataset. Most crucially, MICE is inclined to overfitting when dealing with high-dimensional data (Tang and Ishwaran, 2017). Hence, considering the high-dimensional characteristic of gut microbiome data, MICE might not be the optimal choice for imputation in this context. Secondly, the human gut microbiome involves thousands of genes or microbial features, many of which are irrelevant or noisy, obscuring the relationship between key features and health, leading to overfitting. High-dimensional risk factors increase computation time (Wang et al., 2023a), and complex interrelationships reduce prediction accuracy. In situations with a small sample size, a large number of features can lead to the curse of dimensionality, rendering the data sparse within the feature space. A review (Armstrong et al., 2022) evaluates dimensionality reduction techniques for microbiome data, including principal component analysis (PCA), non-metric multidimensional scaling (nMDS), t-SNE and UMAP. PCA and nMDS are not suitable for handling sparse data, whereas t-SNE and UMAP, although effective in capturing non-linear patterns, are highly sensitive to parameter settings, making their results less reliable and harder to reproduce.Additionally, some studies have also explored nonlinear techniques such as Variational Autoencoders (VAE) (Rezende et al., 2014), which introduce probabilistic generative models and nonlinear transformations to achieve more representative low-dimensional representations (Li et al., 2020). However, these models encounter challenges such as training instability, slow convergence, and the issue of vanishing gradients when dealing with ultra-high-dimensional datasets. Thirdly, individual machine learning models exhibit variable performance across different datasets, resulting in inconsistent predictions and limited generalization (Ansarullah and Kumar, 2019). While deep learning models can enhance prediction accuracy, they require substantial data, computational resources and face challenges in multi-dimensional data processing (Yekkala et al., 2017). Ensemble learning has yielded promising results across various prediction tasks (Peng et al., 2023; Kalaiselvi and Geetha, 2024). Similarly, it has demonstrated efficacy in predicting the risk of IBD (Alfonso Perez and Castillo, 2023). Studies have shown that random forest (RF), as an ensemble learning method, performs exceptionally well in predicting IBD. One study demonstrated that RF model based on laboratory markers exhibit high accuracy in classifying IBD, particularly achieving AUC values of 97% for Crohn’s disease and 91% for ulcerative colitis (Kraszewski et al., 2021). A study developed a RF model using baseline clinical and serological parameters, achieving an AUC of 0.90 to successfully predict CD patients’ response to IFX treatment, outperforming a logistic regression model (Li et al., 2021). Moreover, research by Alfonso Perez and Castillo (2023) further confirmed that RF models excel in handling complex medical data, making them an excellent choice for IBD prediction, outperforming many other commonly used machine learning algorithms. In addition, hyperparameter settings significantly impact ensemble learning model accuracy. Traditional hyperparameter optimization methods like random search (RS) and grid search (GS) are computationally intensive (Hutter et al., 2019), while Bayesian optimization (BO) (Chen et al., 2023) and particle swarm optimization (PSO) (Wang et al., 2023b), and Gray Wolf optimization (GWO) (Mafarja and Mirjalili, 2017) can get trapped in local optima. Direct use of aquila optimizer (AO) (Abualigah et al., 2021) also risks local optima issues.

To address these issues, we proposed the CDORPF, a comprehensive data optimization and risk prediction framework. This framework was divided into two main modules: data optimization and risk prediction. In the data optimization module, we first employed triple optimization imputation (TOI) to impute missing data while preserving the biological characteristics of the gut microbiome data. Next, we introduced the importance-weighted variational autoencoder with integrated evaluation (IWVAE) method, which incorporated feature importance ranking and a comprehensive scoring approach based on VAE, to enhance the dimensionality reduction process by retaining critical features. This resulted in a more complete dataset and a low-dimensional representation, laying a solid foundation for improving algorithm efficiency and accuracy. In the risk prediction module, the optimized data was classified using the RF model, while the improved aquila optimizer (IAO), enhanced with multiple strategies, was employed for global hyperparameter optimization of the RF model. The effectiveness of the CDORPF framework had been validated through multiple comparative experiments.

## 2 Materials and methods

### 2.1 CDORPF

**Figure 1:**
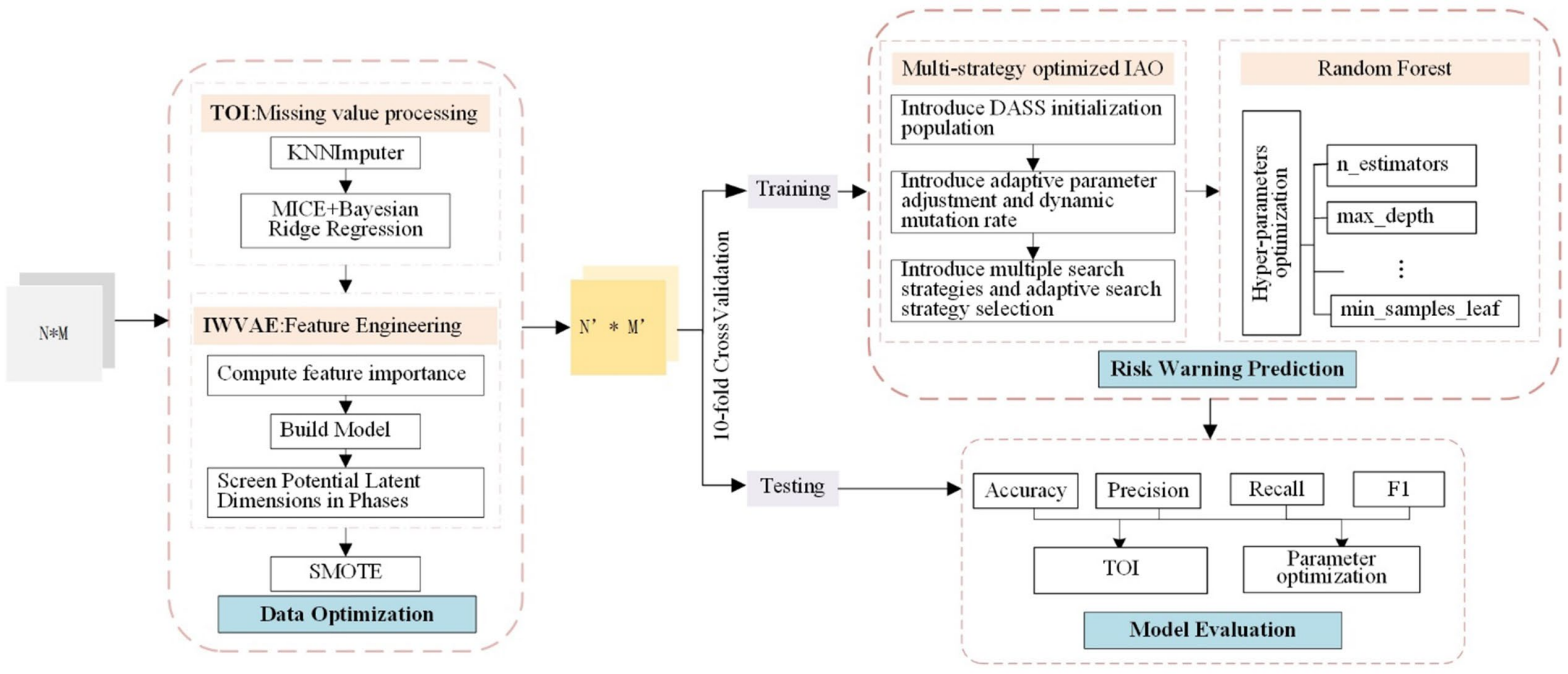
Flow work of CDORPF.

The overall framework structure of CDORPF is illustrated in Figure 1.

Where, the input is a N*M matrix, and the output is a *N*′*M′ matrix. *N* represents the number of original data items, *M* refers to the feature dimension of the data items, and *N*′ and *M*′ denote the sample size and feature dimension, respectively, after data optimization.

The workflow of the model is as follows:

(1) Data optimization: TOI is applied to impute missing data in the original dataset. IWVAE is then utilized for dimensionality reduction, and SMOTE (Feng et al., 2023) is employed to address sample imbalance, ensuring consistency and reliability of the human gut microbiome data while maintaining biological accuracy.
(2) Predictive model construction: Initial parameter ranges for the RF model are set, including n_estimators, max_depth, min_samples_split and min_samples_leaf. IAO optimizes these parameters through iterative searches. Enhancements such as dynamic adjustment Sobol sequence (DASS), adaptive parameter adjustment, and dynamic mutation rates are introduced. The optimized hyperparameters are used to train the RF model, which is then evaluated on a validation set.
(3) Model evaluation: Performance metrics for the predictive model are proposed and compared with classical and widely-used models to assess CDORPF.

### 2.2 Data imputation based on TOI

Gut microbiome data frequently exhibit high-dimensional sparsity (Xie and Lederer, 2021), and multicollinearity may be present among the features (Die et al., 2022). For sparse data, relying solely on KNN often encounters challenges in identifying sufficiently similar neighboring samples, while using MICE alone may fail to capture strong feature correlations. KNN excels at managing locally similar samples and effectively captures local structural characteristics between them, whereas MICE employs regression models that leverage global feature correlations for imputation. By combining KNN and MICE, both local and global information can be leveraged to more comprehensively fill in missing values. Additionally, introducing ridge regression during MICE imputation can effectively reduce model instability caused by multicollinearity, enhancing the robustness of the model and making the imputation results more stable and accurate. Ridge regression achieves this through regularization, which prevents overfitting to noise inherent in sparse data while preserving reasonable correlations among features.

Based on the above three methods, we proposed TOI. TOI not only preserves the dataset’s integrity but also retains the intrinsic structure and relationships within the microbiome data, supporting reliable subsequent analysis and model development.

The process is as follows:

Step 1. Initial imputation: For a dataset *X* containing missing values, the KNN is employed for preliminary imputation.

(1) Distance calculation: The distance between record *i* and other records in the dataset is computed using the appropriate Equation 1.

(1) $$d\left(i,n\right)=\sqrt{\overset{p}{\underset{m=1}{\sum }}{\left(x_{i,m}-x_{n,m}\right)}^{2}}$$

(2) Select nearest neighbors: Based on the calculated distances, select the *k* records that are closest.
(3) Calculate mean: Compute the mean of feature *j* across these *k* nearest neighbors using Equation 2 and use this value to impute the missing data.

(2) $${\overset{\wedge }{x}}_{i,j}=\frac{1}{k}\underset{n\in N_{K}\left(i\right)}{\sum }x_{n,j}$$

where, ${\overset{\wedge }{x}}_{i,j}$ represents the imputed value of feature *j* for record *i*, $N_{k}\left(i\right)$ denotes the set of indices corresponding to the *k* nearest neighbors of record *i*, $x_{n,j}$ refers to the values of feature *j* among these nearest neighbors.

Step 2. Iterative optimization of imputation: Building on the initial imputation, multiple imputation is performed with iterative refinement. In each iteration, a Bayesian ridge regression model is used to predict the imputed values.

(1) Target feature selection: In each iteration, select feature *j* as the target variable, with the remaining features serving as predictors.
(2) Model construction: Using the other feature values $x_{i,\neg j}^{\left(t\right)}$ of record *i*, construct the Bayesian Ridge Regression model $f_{j}$ and compute the regression coefficients $\overset{\wedge }{\beta }$ , as shown in Equation 3.

(3) $$\overset{\wedge }{\beta }={\left(X^{T}X+\lambda I\right)}^{-1}X^{T}y$$

(3) Based on the Equation 4, predictions are made to obtain updated imputed values.

(4) $${\overset{\wedge }{x}}_{i,j}^{\left(t+1\right)}=f_{j}\left(x_{i,\neg j}^{\left(t\right)}\right)+\varepsilon_{i,j}^{\left(t\right)}$$

where, ${\overset{\wedge }{x}}_{i,j}^{\left(t+1\right)}$ represents the imputed value of feature *j* for record *i* in the *t + 1* iteration, $f_{j}$ denotes the regression model for feature *j*, $x_{i,\neg j}^{\left(t\right)}$ refers to all feature values of record *i* excluding feature *j* in the *t* iteration, $\varepsilon_{i,j}^{\left(t\right)}$ is the residual value.

Step 3. Iterative refinement: Repeat the iterative process, selecting each feature for imputation and continuously optimizing the imputed values until convergence is achieved or the maximum number of iterations is reached.

Step 4. Final imputation results: Obtain the final optimized imputation results, filling in all missing values.

In summary, TOI combines the simplicity of KNN, the iterative refinement capability of MICE, and the regularization strength of Bayesian ridge regression. TOI effectively captures both linear and nonlinear relationships in the data, ensuring data integrity and enhancing model prediction performance for more accurate and stable missing data handling.

### 2.3 Data dimensionality reduction based on IWVAE

Building on VAE, we propose IWVAE, which integrates feature importance ranking and a comprehensive scoring mechanism to effectively reduce data dimensionality while maintaining high classification performance.

Step 1. Calculate feature importance: Feature importance scores are computed using RF, and features are ranked accordingly. This approach prioritizes the retention of the most critical features for classification tasks, enhancing the efficiency of the dimensionality reduction process.

Step 2. Definition of VAE: The encoder maps high-dimensional data into a low-dimensional latent space, while the decoder reconstructs the high-dimensional data from this latent representation. The encoder outputs include the latent mean μ and the latent log-variance $\log\left(\sigma^{2}\right)$ , with the KL divergence loss term constraining the distribution in the latent space as shown in Equation 5.


(5) $$KL\left(q\left(z|x\right)||p\left(z\right)\right)=-\frac{1}{2}\overset{J}{\underset{j=1}{\sum }}\left(1+\log\left(\sigma^{2}\right)-\mu_{j}^{2}-\sigma_{j}^{2}\right)$$

The decoder reconstructs the data using the latent variable *z*, and the reconstruction error is computed based on the following Equation 6:


(6) $$\mathrm{Reconstruction}\;\mathrm{error}=\frac{1}{N}\overset{N}{\underset{i=1}{\sum }}x_{i}-{\overset{\wedge }{x_{i}}}^{2}$$

where, $x_{i}$ represents the original data, $\overset{\wedge }{x_{i}}$ represents the reconstructed data, N denotes the sample size.

Step 3. Preliminary screening stage: Features are selected at intervals of 1/10 of the total dimensionality. The trained VAE model is used to calculate reconstruction errors, and RF is trained on the dimensionally reduced data. Classification accuracy is evaluated through cross-validation. By balancing reconstruction error and classification accuracy, the introduction of a comprehensive score avoids bias and overfitting, enabling a more thorough model evaluation and ensuring an optimal balance between preserving data features and predictive capability. Both the reconstruction error and classification accuracy are standardized using specific Equations 7 8, and a comprehensive score is computed using Equation 9. The optimal latent dimensions are then recorded.


(7) $$SRE=\frac{\mathrm{Reconstruction}\;\mathrm{error}-\min\left(\mathrm{reconstruction}\;\mathrm{error}\right)}{\max\left(\mathrm{Reconstruction}\;\mathrm{error}\right)-\min\left(\mathrm{Reconstruction}\;\mathrm{error}\right)}$$

(8) $$SMO=\frac{\mathrm{Model}\;\mathrm{accuracy}-\min\left(\mathrm{Model}\;\mathrm{accuracy}\right)}{\max\left(\mathrm{Model}\;\mathrm{accuracy}\right)-\min\left(\mathrm{Model}\;\mathrm{accuracy}\right)}$$

(9) $$\mathrm{Combined}\;\mathrm{score}=SRE+\left(1-SMO\right)$$

Step 4. Refined screening phase: Conduct a more detailed screening around the optimal latent dimensions identified in the preliminary screening phase. All steps from the preliminary screening are repeated within this refined range to ensure precision.

### 2.4 IAO-RF risk prediction model construction

#### 2.4.1 IAO

The traditional AO initializes the population randomly, which may result in insufficient exploration during the early stages and an increased risk of becoming trapped in local optima. Furthermore, its fixed parameter selection and singular search strategy can cause an imbalance between global exploration and local exploitation. To overcome these limitations, IAO incorporates multiple strategies to enhance AO, achieving a balance between local and global optimization while improving search efficiency. The following optimizations have been implemented:

(1) DASS is employed to initialize the population, enhancing the diversity of the initial population.

The Sobol sequence is a quasi-random sequence used to generate low-discrepancy samples. The DASS refines this by incorporating feature importance information to adjust the search space dynamically, overcoming the limitation of the traditional Sobol sequence, which cannot adapt to problem-specific characteristics.

a Calculate feature importance: The importance of each feature is calculated using a baseline model, as described in Equation 10.

(10) $${\mathrm{importance}}_{i}=\frac{1}{n_{\mathrm{trees}}}{\overset{n_{\mathrm{trees}}}{\underset{t=1}{\sum }}}_{\Delta }{\mathrm{Gini}}_{t}\left(i\right)$$

where, $\Delta^{{\text{Gini}}_{t}}(i)$ represents the contribution of feature *i* to the Gini index in the *t*-th tree.

b Dynamic adjustment of search space: The search space is adjusted based on feature importance. If a feature’s importance exceeds a certain threshold, its search range is expanded as specified in Equation 11.

(11) $${\mathrm{bounds}}_{j}=\{\begin{array}{c} \left[x_{\min,j},x_{\max,\mathrm{high},j}\right]\\ \left[x_{\min,j},x_{\max,j}\right] \end{array}\begin{array}{cc} & \mathrm{if}\;{\mathrm{importance}}_{j}>\theta \\ & \mathrm{if}\;{\mathrm{importance}}_{j}\leq \theta \end{array}$$

where, θ is a predefined threshold.

c Sobol sequence generation: The Sobol sequence is used to generate uniformly distributed points, as defined in Equation 12.

(12) $$u=\mathrm{Sobol}\left(d,N\right)$$

where, *u* is a *d × N* matrix, *d* represents the dimensionality of the hyperparameters, *N* refers the population size.

d Mapping to the dynamically adjusted search space: Using Equation 13, the values from the Sobol sequence in the range of [0, 1] are mapped to the dynamic adjustment range of each parameter.

(13) $$x_{ij}=x_{\min,j}+u_{ij}\cdot \left(x_{\max,j}-x_{\min,j}\right)$$

where, $x_{ij}$ represents the *j*-th parameter of the *i*-th individual, $x_{\min,j}$ and $x_{\max,j}$ denote the minimum and maximum ranges of the *j*-th parameter.

**Figure 2:**
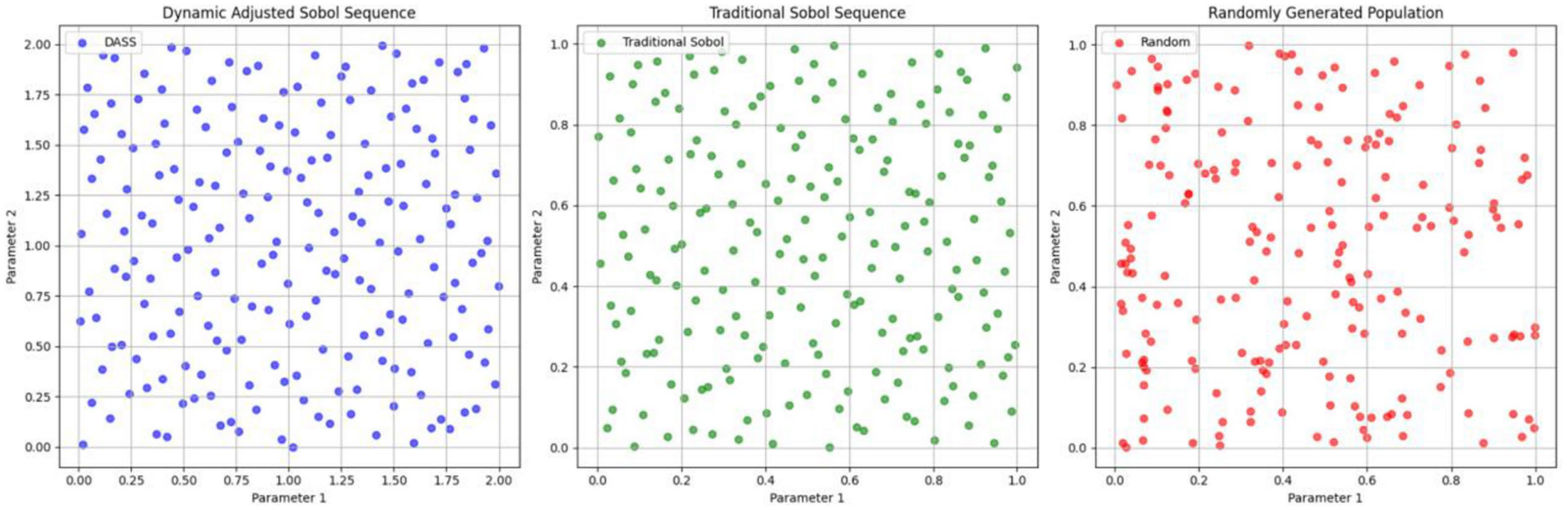
Comparison of population initialization.

Figure 2 shows the distribution of a 2D initial population of size 200 generated using the DASS, the traditional Sobol sequence, and random generation methods. It is evident that the population generated by the dynamically adjusted Sobol sequence is more uniformly distributed, providing broader coverage of the solution space. Notably, within the parameter ranges of higher feature importance, this method maintains better population diversity, which can enhance the optimization speed and convergence accuracy of the algorithm.

(2) Adaptive parameter adjustment and dynamic mutation rates are introduced to adaptively modify the search range and mutation rates at different stages of the optimization process. This effectively balances global exploration and local exploitation, thereby enhancing overall optimization performance.

a Adaptive parameter adjustment: As shown in the Equation 14, parameters are dynamically adjusted based on the number of iterations. This approach strengthens global exploration in the early iterations and enhances local exploitation in the later stages, preventing premature convergence.

(14) $$\alpha =\frac{\max\_\mathrm{iterations}-\mathrm{iteration}}{\max\_\mathrm{iterations}}$$

b Dynamic mutation rate: As shown in the Equation 15, the mutation rate is dynamically adjusted based on the number of iterations, enhancing population diversity and preventing premature convergence.

(15) $$\mathrm{mutation}\_\mathrm{rate}=0.1\times \left(1-\frac{\mathrm{iteration}}{\max\_\mathrm{iterations}}\right)$$

(3) Incorporate a position update strategy, as different strategies can facilitate exploration and exploitation during the optimization process.

- Exploration strategy: The position of individuals is updated using the current best individual ( $x_{\mathrm{best}}$ ) as a reference point. A wide range of movement is achieved through a random factor (rand) and an adaptive parameter ( α ), expanding the search space and enhancing global exploration capabilities. As shown in the Equation 16:

(16) $$x_{i}^{\mathrm{new}}=x_{i}+\alpha \cdot \mathrm{rand}\cdot \left(x_{\mathrm{best}}-x_{i}\right)$$

where, $x_{i}^{\mathrm{new}}$ represents the new position of individual *i*, $x_{i}$ is the current position of individual *i*, α is an adaptive parameter that gradually decreases with the number of iterations, rand is a random number between 0 and 1.

b Exploitation strategy: The position of individuals is updated using the current worst individual ( $x_{\mathrm{worst}}$ ) as a reference point. Local exploitation is achieved through a random factor (rand) and an adaptive parameter ( α ), enhancing local search capabilities. This allows for finer exploration within the current search region, preventing premature convergence to local optima. As shown in the Equation 17:

(17) $$x_{i}^{\mathrm{new}}=x_{i}+\alpha \cdot \mathrm{rand}\cdot \left(x_{\mathrm{worst}}-x_{i}\right)$$

c Levy flight: Individual $x_{i}$ approaches the current best individual ( $x_{\mathrm{best}}$ ) using a random step length $\mathrm{Levy}\left(\beta \right)$ based on the Levy distribution and an adaptive parameter ( α ), as shown in Equations 18 19. This strategy helps to overcome the limitations of local optima and further enhances global exploration capabilities. The formula is as follows:

(18) $$x_{i}^{\mathrm{new}}=x_{i}+\alpha \times \mathrm{Levy}\left(\beta \right)\times \left(x_{i}-x_{\mathrm{best}}\right)$$

(19) Levyβ=uv1β

where, *u* and *v* are random variables that follow a normal distribution, and $x_{\mathrm{mean}}$ is a parameter of the Levy distribution.

d Gradual convergence strategy: In the middle to late stages of the optimization process, individuals gradually converge toward the population mean, as shown in Equation 20. This approach balances global exploration and local exploitation, leading to a gradual convergence. The formula is as follows:

(20) $$x_{i}^{\mathrm{new}}=x_{i}+\alpha \cdot \mathrm{rand}\cdot \left(x_{i}-x_{\mathrm{mean}}\right)$$

(4) Introduce diversity measurement and adaptive strategy selection to dynamically adjust optimization strategies, enabling the algorithm to better balance exploring new solution spaces and optimizing the current solution space.

IAO dynamically selects different search strategies at various optimization stages based on population diversity. When diversity is high, the algorithm favors the exploration strategy and Levy flight to expand the search space. Conversely, when diversity is low, it leans toward the exploitation strategy and gradual convergence strategy to optimize the current solution. The standard deviation across each dimension of the population is calculated using Equation 21 to measure population diversity, effectively reflecting the distribution of the population within the search space.


(21) $$D=\frac{1}{n}\overset{n}{\underset{j=1}{\sum }}\sigma_{j}$$

where, *D* represents the population diversity, *n* is the number of dimensions, and $\sigma_{j}$ is the standard deviation of the population in the *j*-th dimension.

#### 2.4.2 The algorithmic process of IAO-optimized RF

The key steps in optimizing RF with IAO are as follows:

Step 1: Initialize parameters: population size, parameter dimensions (dim), parameter ranges (x_min and x_max) and maximum iterations (max_iterations).

Step 2: Define the fitness function.

Step 3: Initialize the population using DASS.

Step 4: Iterative optimization process:

- Calculate the population mean ( $x_{\mathrm{mean}}$ ) and the worst individual ( $x_{\mathrm{worst}}$ ).
- Calculate adaptive adjustment parameters based on the Equation 14.
- Compute the dynamically adjusted mutation rate using the Equation 15.
- Retain the current best individual ( $x_{\mathrm{best}}$ ) using an elite strategy.
- Execute the search strategy for each individual:

Select the search strategy based on population diversity.
Implement exploration, exploitation, Levy and gradual convergence strategies.

- Calculate the fitness of new individuals and update the population.

Step 5: Output the optimal parameters to construct the IAO-RF model for prediction tasks.

### 2.5 Model performance measures

Using stratified random sampling to maintain class distribution consistency, the dataset was divided into two subsets: 80% for the training dataset and 20% for the test dataset. The training dataset was used to train the machine learning models, and the test dataset was used to evaluate model performance. Ten-fold cross-validation was performed. Based on the test dataset and the model predicted target variables, five statistical measures were used to evaluate the model performance: accuracy, precision, recall, F1-score and AUC.

### 2.6 Baseline methods

To demonstrate the effectiveness of the proposed model, we compared it with several widely used models across different categories. For evaluating the effectiveness of various imputation methods, we employed classifiers such as logistic regression (LR), SVM, MLP, XGBoost, LightGBM and RF. To assess the performance of dimensionality reduction methods, we compared PCA, VAE and IVAE. For evaluating parameter optimization methods, we compared RS, GS and BO.

## 3 Results

### 3.1 Data selection

The experimental data used in this study is from the Inflammatory Bowel Disease Multi’omics Database (IBDMDB) within the Integrated Human Microbiome Project (iHMP) (Lloyd-Price et al., 2019). Microbial community structure and diversity were analyzed using 16S rRNA gene sequencing, specifically targeting the V4 region. This gut microbiome dataset comprises 178 participant records, in which 137 with IBD and 41 without. Each record consists of 983 fields. Notably, one field serves as an indicator for the presence or absence of IBD, while the remaining 982 fields represent an array of microbial features. However, many feature values in most samples are either close to zero or exactly zero, leading to a sparse data distribution in high-dimensional space. Despite this overall sparsity, some samples exhibit high abundance in specific feature dimensions, creating locally dense regions. Additionally, approximately 491 features have missing values, with missing rates ranging from 0.56% to 9.55%, and an average missing rate of 5.27%.

In summary, this dataset is characterized by high-dimensional sparsity, the presence of missing values, differences in class distribution, and local density regions.

### 3.2 Data optimization

#### 3.2.1 Data imputation based on TOI and effectiveness analysis

##### 3.2.1.1 Validation of the rationale for using TOI

Data missingness can be classified as missing completely at random (MCAR), missing at random (MAR) and not missing at random (NMAR). To validate the rationale for using the TOI for data imputation, the first step is to identify the type of missing data within the dataset.

###### 3.2.1.1.1 Analysis of missing data types in the dataset

A correlation matrix was used to analyze the types of missing data by displaying the correlations between missing values across different features. The matrix colors range from blue (negative correlation) to red (positive correlation). Blue indicates that when one feature is missing, another is likely to be present, while red suggests that missing values in two features tend to occur together.

**Figure 3:**
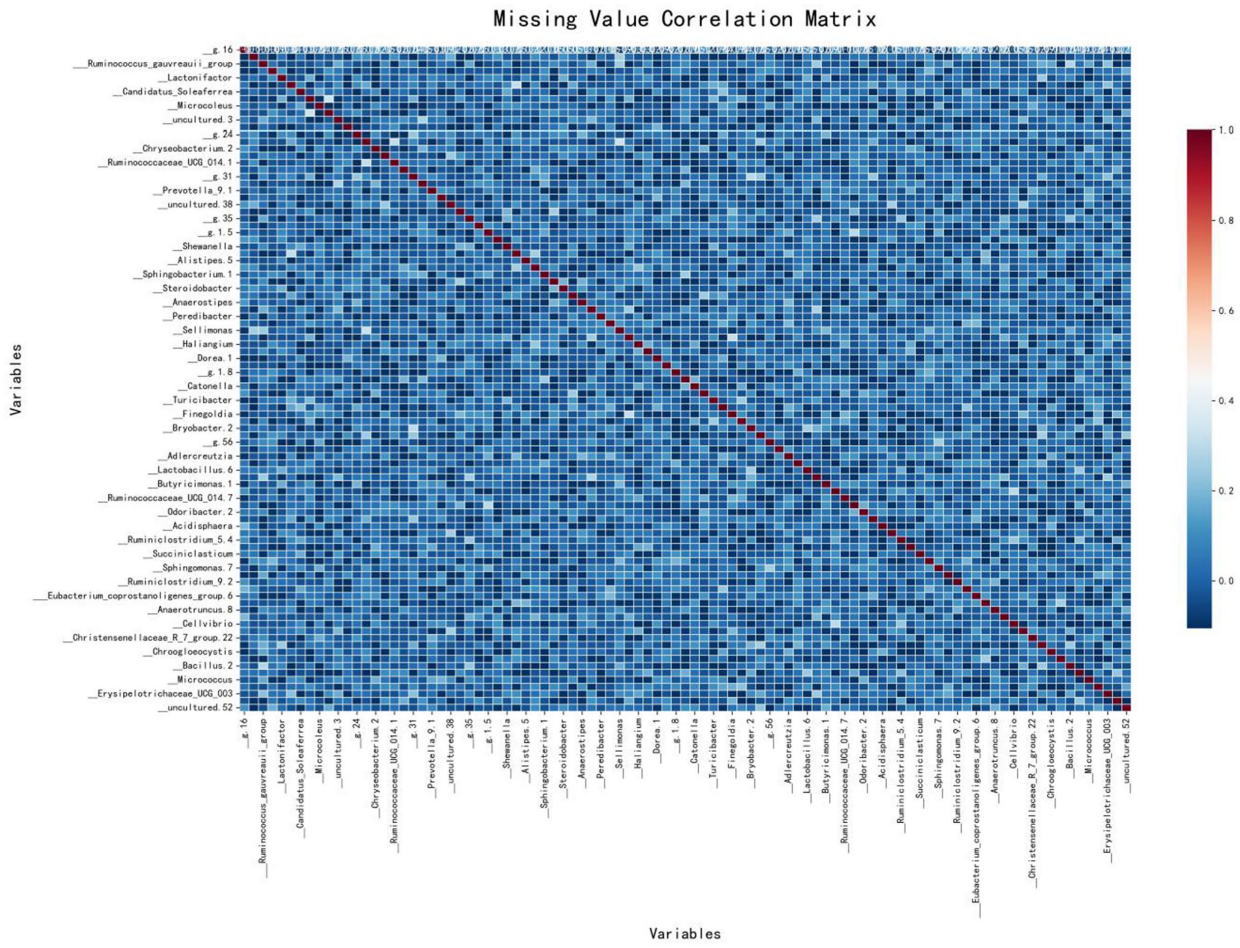
Missing value correlation matrix.

As shown in Figure 3, the correlation matrix of missing values in the IBD dataset is predominantly blue, indicating no significant correlation between the missing values of different variables. This suggests that the missing values in this dataset are likely to be MAR, meaning that the missingness of certain variables may be related to other observed variables, but not to the missing data itself. The red areas are mainly along the diagonal, showing that each variable is perfectly correlated with its own missing values, which is expected. Therefore, this figure suggests that most variables have independent missing values, indicating the missingness mechanism in this dataset is likely MAR.

###### 3.2.1.1.2 Analysis of grouped statistical analysis and hypothesis testing results

Grouped statistical analysis provides a statistical comparison between missing and non-missing groups by analyzing their means and standard deviations. Hypothesis testing, through *t*-tests on key variables, evaluates whether significant differences exist between missing values and other variables. Selected experimental results are shown in Table 1.

**Table 1:** Results of grouped statistical analysis and hypothesis testing.

Feature	Variable	Mean (missing)	Mean (non-missing)	*t*-statistic	*p*-value
_Tepidimonas	_Bacteroides	3348.25	2366.95679	−1.136126196	0.257484265
_Tepidimonas	_Bacteroides.6	2147.166667	1618.566265	−0.617270031	0.537854396
_Prevotella	_Bacteroides	1857.416667	2477.388889	0.716181482	0.474850527
_Prevotella	_Faecalibacterium.2	1785.666667	2335.963855	0.737912748	0.461550206
_Prevotella	_Escherichia_Shigella	447.6666667	626.9036145	0.300885428	0.763857015
_Belnapia	_Bacteroides.6	434	1661.096045	0.426897256	0.669975915
_Belnapia	_Dialister.2	318	147.3619632	−0.662256296	0.508748001
_Belnapia	_Lachnoclostridium.1	272	86.81920904	−0.781271529	0.435692463

Most variables show no significant differences between missing and non-missing samples (*p*-value >0.05), indicating a weak association with missingness. However, some features exhibit significant differences (*p*-value <0.05), suggesting a potential relationship, while others show near-significant differences (*p*-value close to 0.05), indicating a possible but not conclusive association. These findings suggest that the majority of missing data in the dataset are not significantly related to other variables, implying a likely MAR.

The combined results of both experiments confirm that the missing data mechanism in the IBD dataset is MAR, supporting the rationale for using TOI for imputation.

##### 3.2.1.2 TOI-based data imputation

###### 3.2.1.2.1 Imputation results of the dataset

**Figure 4:**
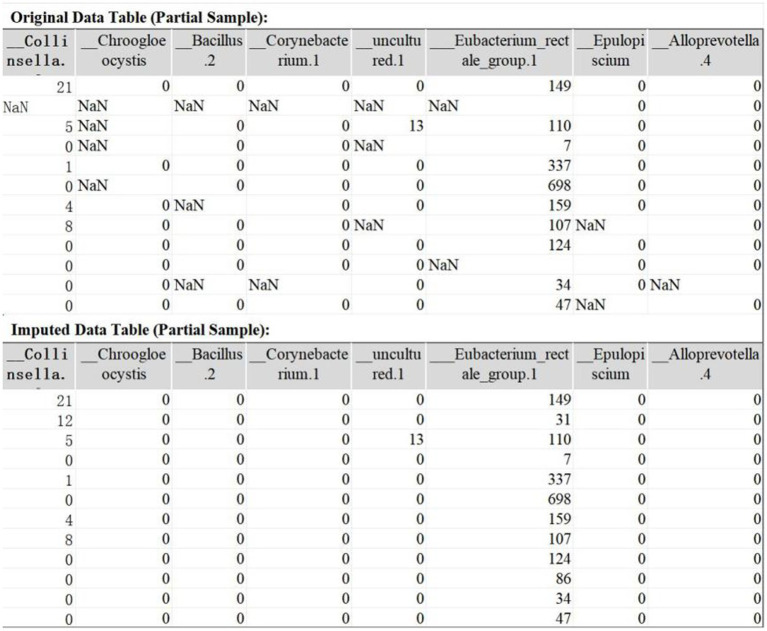
Example of data tables before and after imputation (partial sample).

The original IBD dataset contains 491 features with missing values, with missing rates ranging from 0.56 to 9.55%. TOI successfully imputed all missing values. A comparison of the dataset before and after imputation is presented in Figure 4.

###### 3.2.1.2.2 Comparison of distributions before and after imputation

**Figure 5:**
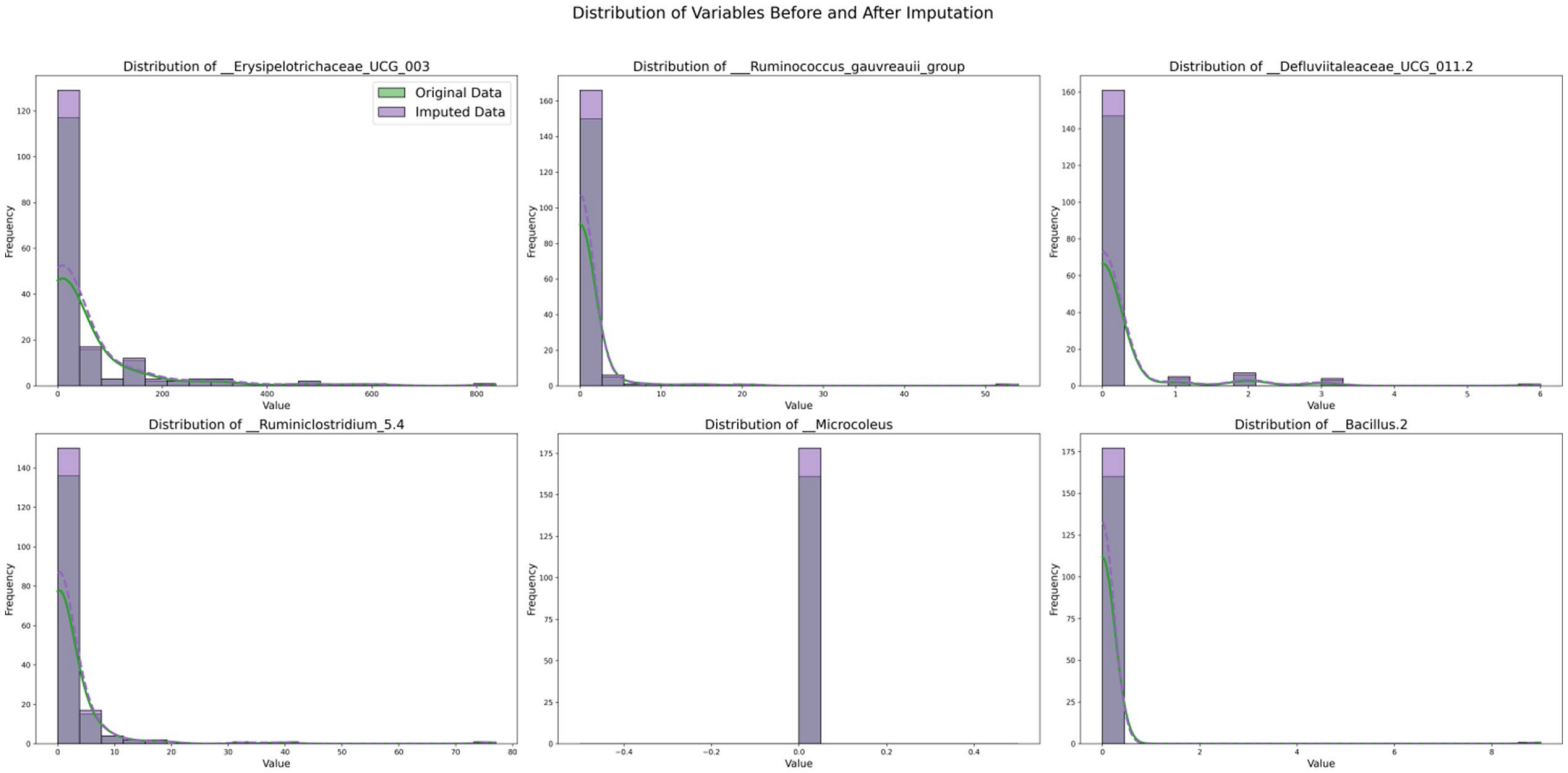
Distribution of variables before and after imputation.

By comparing the data distributions before and after TOI imputation, the impact of TOI on the data was visually assessed, validating the effectiveness and fidelity of the imputed data. If the post-imputation distribution aligns with the original data, it indicates that the imputation method is appropriate. Selected experimental results are shown in Figure 5.

Figure 5 illustrates the comparative distributions of variables with substantial missing rates, both prior to and following imputation. Each subplot displays the distribution of an individual variable, where the original data are depicted in green and the imputed data in purple. The histograms illustrate the frequency distribution of the variables, and by contrasting the green and purple histograms, one can distinctly perceive the alterations in data frequency across diverse value ranges before and after imputation. The kernel density estimation (KDE) curves offer a smooth estimation of the probability density, further highlighting the distribution tendencies of the data.

The outcomes imply that the distribution of the variable__Erysipelotrichaceae_UCG_003 remains highly consistent before and after imputation, indicating that the imputed data effectively preserves the traits of the original data. For the variable__Defluviitaleaceae_UCG_011.2, the imputed data fills the sparse regions of the original distribution, leading to a more smoother distribution, which attests to the efficacy of the imputation technique. On the whole, the imputed distributions (purple) closely resemble the original distributions (green) for the majority of variables, as evidenced by the similarity in the histograms and KDE curves in each subplot. These findings indicate that our imputation methodology effectively preserves the key distributional properties of the original dataset. For variables exhibiting long-tailed distributions, the imputed data preserve this characteristic, thereby underscoring the efficacy of the imputation technique in addressing sparsity.

##### 3.2.1.3 Evaluation of imputation results validity

**Figure 6:**
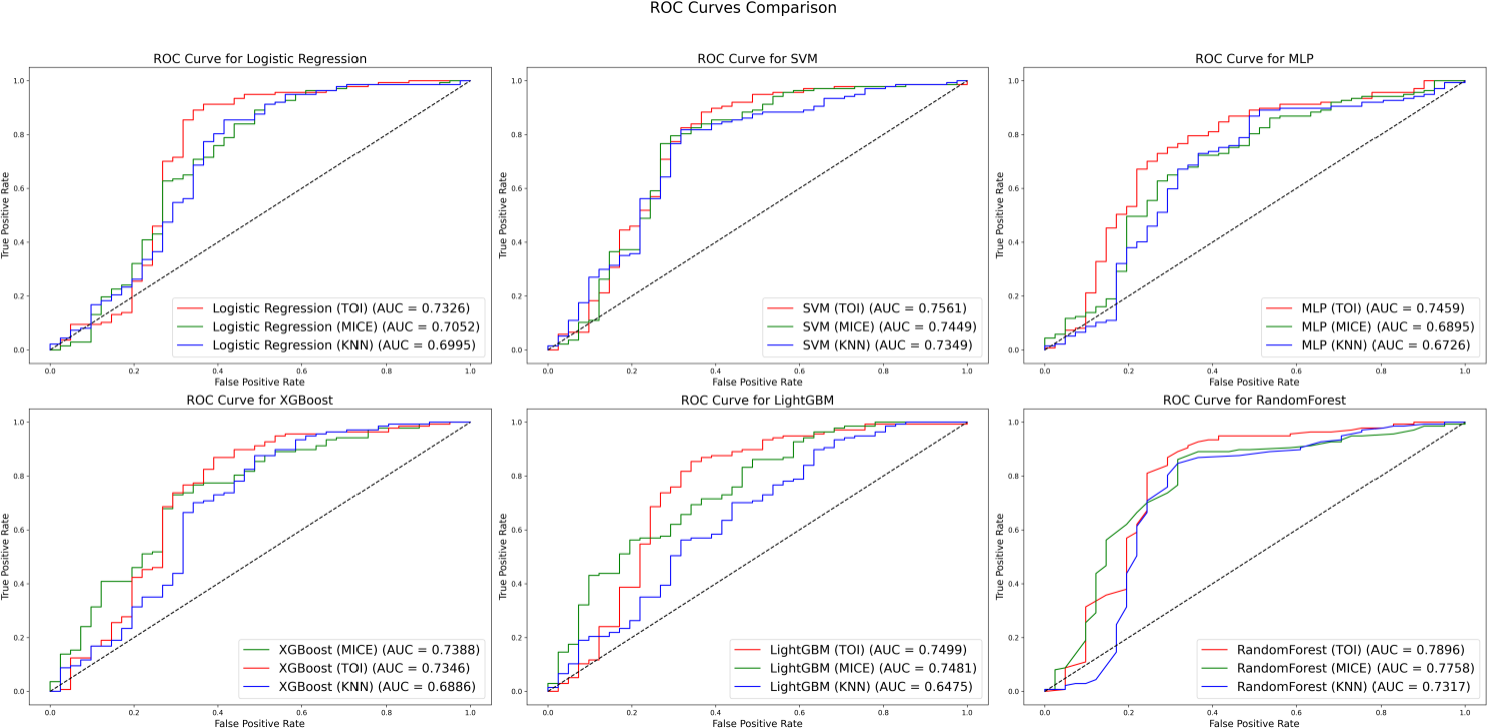
ROC curves comparison.

To further validate the effectiveness of TOI, comparative experiments were conducted using datasets imputed with KNN, MICE and TOI. Classifier models such as LR, SVM, MLP, XGBoost, LightGBM and RF were selected for analysis. The experimental results are presented in Figure 6.

Figure 6 compares the performance of different imputation methods across various classifier models: red for TOI, green for MICE and blue for KNN. The results show that the TOI imputation method achieves the highest AUC values in the evaluations of LR, SVM, MLP, LightGBM and RF models, indicating superior classification performance. Although MICE slightly outperforms TOI in the XGBoost, the AUC difference is only 0.0042. Overall, the TOI imputation method provides better classification performance, demonstrating excellent generalization capability and robustness.

Furthermore, when comparing the performance of various imputation methods, the RF model stands out as particularly exceptional across the entire dataset. With the TOI, the RF achieves an AUC of 0.7896, which is higher than that of most other models using the same method. For example, the AUC for the SVM, LR and XGBoost models under the TOI are 0.7561, 0.7326 and 0.7346, respectively. Additionally, even with the MICE and KNN, the RF’s AUC values remain highly competitive, outperforming those of most other models. These results indicate that the RF model not only maintains a high level of classification performance when handling imputed data but also demonstrates greater stability across different imputation methods, making it the optimal choice for this dataset.

#### 3.2.2 Data dimensionality reduction based on IWVAE and effectiveness analysis

##### 3.2.2.1 Selection of data dimensions

**Figure 7:**
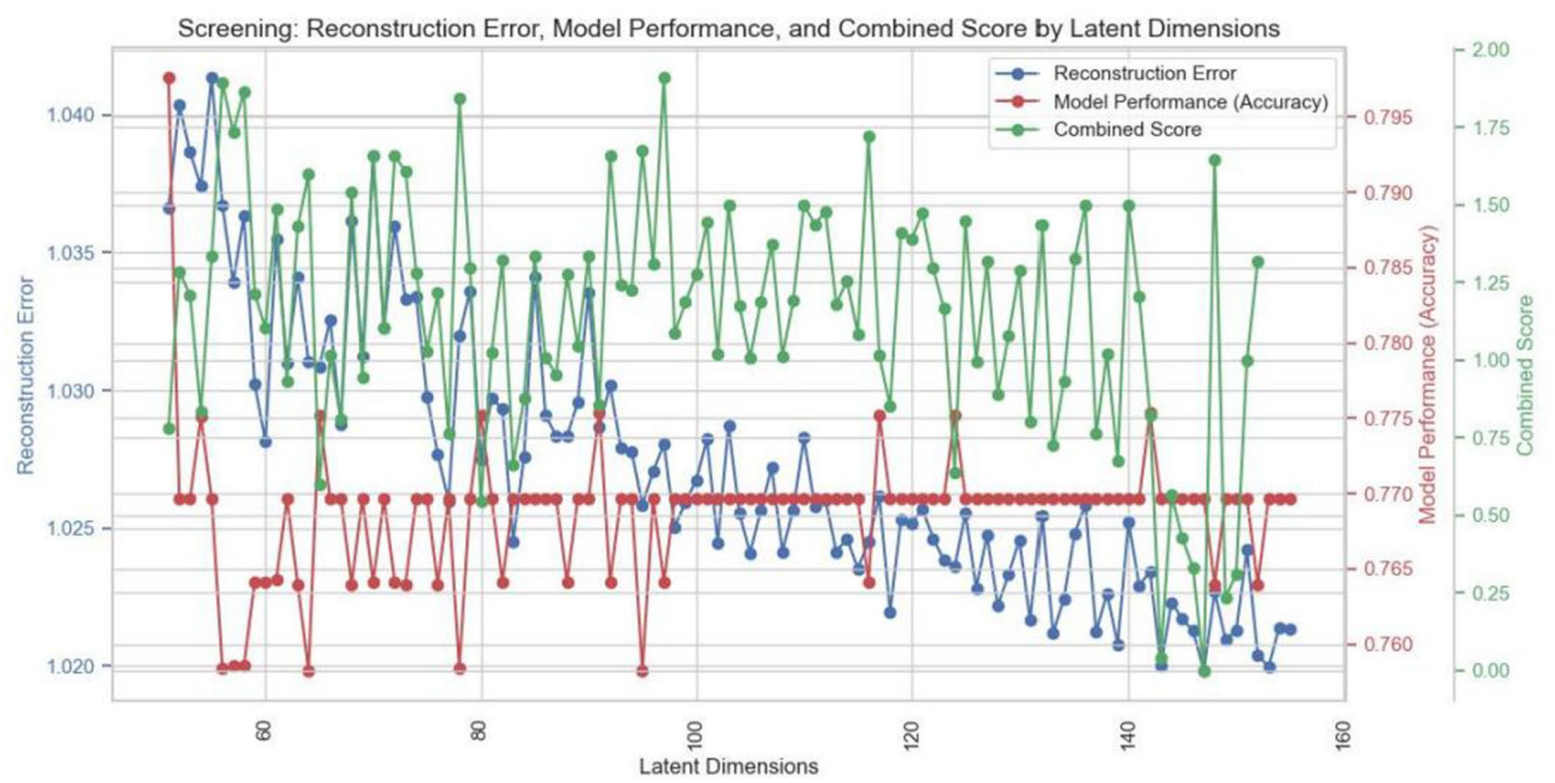
Optimal latent dimension selection.

Figure 7 shows the impact of latent dimensions on reconstruction error (blue curve), model performance (red curve) and the combined score (green curve) during the refinement phase. The blue curve, representing reconstruction error, exhibits fluctuations but generally trends downward as the number of dimensions increases, indicating that data after dimensionality reduction effectively reconstructs the original dataset. The red curve illustrates variations in model performance, with accuracy oscillating between 0.765 and 0.795, suggesting that predictive capability remains relatively stable across different dimensionalities. The green curve, reflecting the combined score, demonstrates significant variability—particularly at higher dimensions—indicating that the dimensionality reduction method is more effective at specific levels. From this figure, it is evident that while both reconstruction error and model performance remain relatively stable as latent dimensions vary, the fluctuations in combined scores imply substantial changes in overall model efficacy across different dimensions. Based on this analysis, 147 dimensions were identified as optimal for achieving an ideal balance between minimizing reconstruction error and enhancing model performance.

##### 3.2.2.2 Analysis of the effectiveness of dimensionality reduction

To further validate the effectiveness of IWVAE, comparative experiments were conducted using the imputed dataset as the experimental dataset. The RF model was applied to the full feature set, as well as to the feature sets reduced by PCA, VAE and IWVAE. The experimental results are presented in Table 2.

**Table 2:** Results of IBD risk prediction using different dimensionality reduction strategies.

Method	Accuracy	Precision	Recall	F1-score
Full features	0.7863	0.6888	0.7863	0.7098
PCA	0.7980	0.7817	0.7980	0.7711
VAE	0.7696	0.5926	0.7696	0.6695
IWVAE	0.8369	0.8459	0.8369	0.8146

As shown in Table 2, IWVAE outperforms all other methods across all metrics, with accuracy, precision, recall and F1 scores all exceeding 0.8. This demonstrates that IWVAE has superior feature extraction capabilities when handling this type of data, significantly enhancing the overall performance of the model.

Following data optimization, the dataset comprises 274 samples with 147 features and no missing values. Of these, 137 samples are from IBD patients, while the remaining 137 are from healthy controls.

### 3.3 IBD risk prediction based on the IAO-RF

#### 3.3.1 Sensitivity analyses

The primary role of sensitivity analysis is to evaluate the model’s response to variations in input parameters and to help identify key parameters that have the most significant impact on the model’s output. Sensitivity analysis allows us to understand how the model performs under different parameter settings, determining its stability and robustness, thereby preventing overfitting or underfitting.

**Figure 8:**
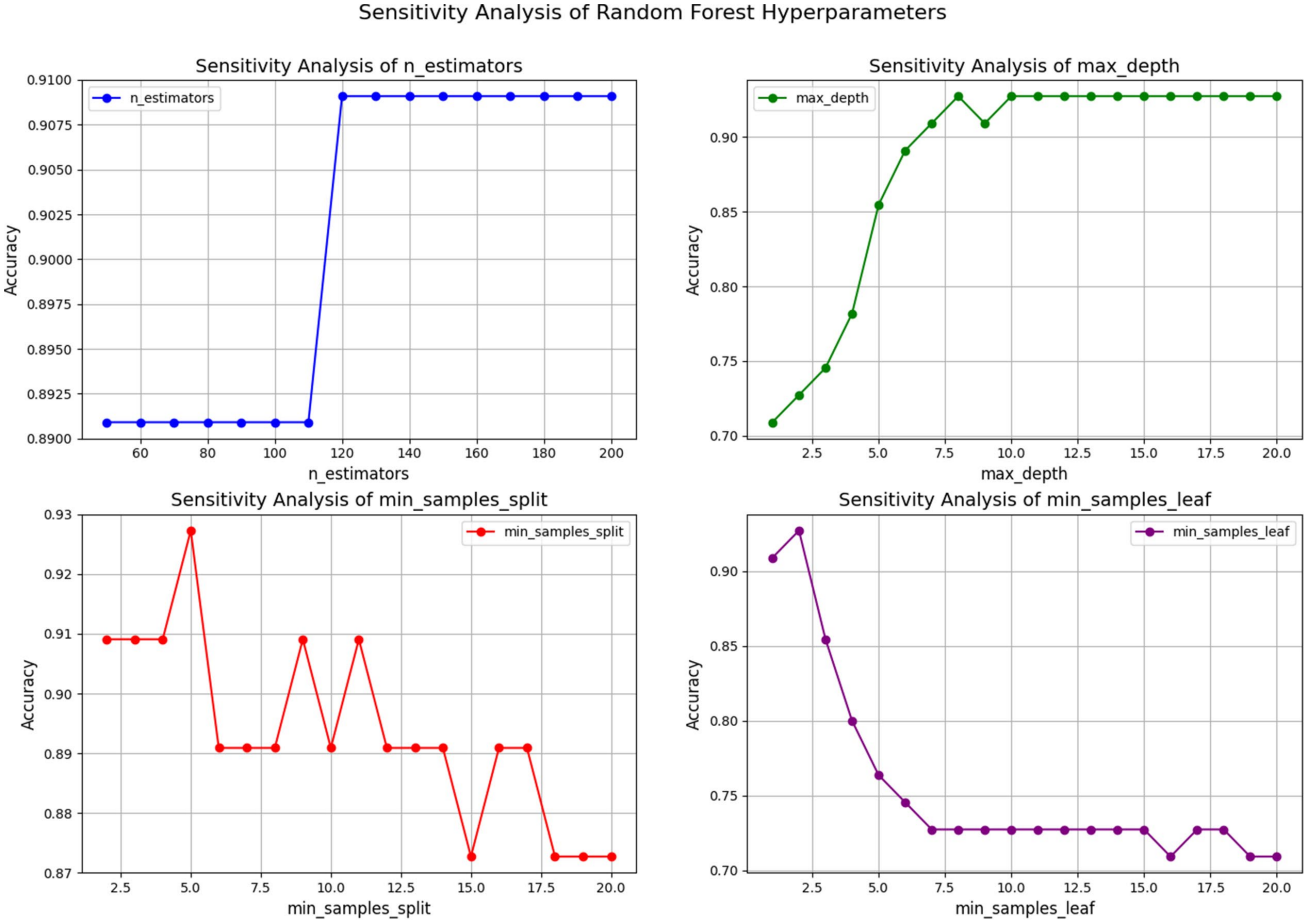
Sensitivity analysis of RF hyperparameters.

Figure 8 shows the sensitivity analysis of four hyperparameters on the accuracy of the RF model. The top-left plot indicates that increasing the n_estimators from 100 to 120 significantly improves accuracy, which then stabilizes beyond 120 trees, suggesting limited benefits from adding more trees after this point. The top-right plot demonstrates that increasing the max_depth improves accuracy until it plateaus at a depth of 10, implying that deeper trees capture data complexity better, but further increases do not boost accuracy. The bottom-left plot reveals that setting min_samples_split around 5 achieves optimal accuracy, with further increases leading to a decline, indicating that too high a threshold for node splitting can cause underfitting. Lastly, the bottom-right plot indicates that setting min_samples_leaf to 2 maximizes accuracy; further increases in this value negatively impact accuracy, implying that a higher number of samples in leaf nodes may reduce model complexity and predictive power.

In summary, hyperparameter selection is crucial for RF performance. Proper tuning of parameters like n_estimators and max_depth can significantly enhance accuracy, while excessively high values for min_samples_split and min_samples_leaf can hinder performance. Consequently, careful adjustment based on the dataset is essential for optimal results.

#### 3.3.2 Parameter settings

The RF model includes multiple hyperparameters within a large parameter space. In this experiment, IAO was employed to optimize four key hyperparameters: n_estimators, max_depth, min_samples_split and min_samples_leaf. The optimization results are presented in Table 3.

**Table 3:** The hyper-parameters tuning results of IAO-optimized RF.

Hyper-parameter	Description	Parameter range	Tuning result
n_estimators	The number of trees in the forest	[10, 200]	200
max_depth	The maximum depth of the trees	[1, 50]	9
min_samples_split	The minimum number of samples required to split an internal node	[2, 10]	2
min_samples_leaf	The minimum number of samples required at a leaf node	[1, 5]	1

Comparative experiments and model evaluation

To further validate the effectiveness of IAO, a comparative experiment was conducted using the processed dataset. The RF model was optimized using no optimization, RS, GS, BO and IAO. The experimental results are presented in Table 4.

**Table 4:** Results of different parameter optimization methods.

Method	Accuracy	Precision	Recall	F1-score
No optimization	0.8727	0.8731	0.8727	0.8726
BO	0.8545	0.8626	0.8545	0.8540
GS	0.8727	0.8775	0.8727	0.8725
RS	0.8909	0.8996	0.8909	0.8905
IAO	0.9043	0.9084	0.9043	0.9040

As shown in Table 4, IAO outperformed all other methods on all metrics, achieving accuracy, precision, recall and F1 scores above 0.9, significantly surpassing other approaches. In contrast, BO and GS failed to notably improve model performance, and although RS provided some enhancement, it remained inferior to IAO. Overall, IAO exhibited a distinct advantage in parameter optimization, leading to enhanced model accuracy, precision, recall and F1 scores.

### 3.4 Effectiveness of CDORPF

To further validate the effectiveness of CDORPF, we conducted a comparative experiment. This experiment compared commonly used machine learning models with CDORPF and the results are presented in Table 5.

**Table 5:** Effectiveness of CDORPF.

Model	Accuracy	Precision	Recall	F1-score
XGBoost	0.7500	0.6481	0.6607	0.6535
LightGBM	0.7222	0.3889	0.5000	0.4375
CatBoost	0.7778	0.6563	0.5893	0.6000
SVM	0.7778	0.3889	0.5000	0.4375
Mice + RF	0.8333	0.8627	0.8333	0.7914
Mice + SVM	0.7778	0.6049	0.7778	0.6806
CDORPF	0.9043	0.9084	0.9043	0.9040

Table 5 clearly demonstrates that the CDORPF framework significantly outperforms other commonly used machine learning models across all evaluation metrics, confirming its superior accuracy and reliability. Moreover, CDORPF exhibits a notable level of consistency in accuracy, precision, recall and F1-score, which is critically important for practical applications.

## 4 Discussion and interpretation

We developed a framework named CDORPF to address the issue of missing values in microbiome data, transforming high-dimensional microbiome profiles into low-dimensional representations and constructing classification models based on these representations.

In the initial phases of this research, our primary objective was data imputation, as most machine learning algorithms are not designed to effectively handle missing values, which can lead to bias. We explored traditional KNN and MICE methods for imputation; however, the results were suboptimal. To preserve the inherent structure and relationships within microbiome data, we proposed the TOI method that integrates KNN, MICE and Bayesian ridge regression. By analyzing the types of missing data present in the IBD dataset, we validated the rationale behind the TOI method and successfully imputed all missing values while maintaining internal structural integrity. As illustrated in Figure 5, experimental results indicate that the distribution of imputed data closely aligns with that of original data—demonstrating that TOI enhances completeness while preserving critical features—thereby laying a solid foundation for subsequent analyses and model development.

Building upon this groundwork, we conducted dimensionality reduction experiments since high dimensionality in 16S rRNA data introduces noise detrimental to downstream predictions. Our proposed IWVAE method outperformed PCA and VAE by effectively reducing dimensions while retaining essential features. As presented in Table 2, IWVAE achieved superior performance across metrics such as accuracy, precision, recall and F1 score—significantly enhancing overall model efficacy and showcasing its exceptional capability in feature extraction.

In our model optimization experiments involving global hyperparameter tuning using IAO-RF demonstrated notable advantages over RS, GS and BO (Table 4). This further substantiates the effectiveness of IAO optimization strategies in improving model performance particularly when dealing with complex datasets characterized by enhanced accuracy and stability.

In summary, our CDORPF framework exhibits significant strengths in addressing issues related to incompleteness, high dimensionality and sparsity within microbiome datasets. As evidenced by Table 5, CDORPF surpasses traditional machine learning models across all evaluated metrics offering improved accuracy alongside consistency thus affirming its potential applicability within real-world scenarios.

By adeptly integrating components such as data imputation, dimensionality reduction and risk prediction, the CDORPF framework effectively confronts challenges associated with microbiome information. Future investigations could further validate this framework’s robustness on larger more intricate datasets whilst exploring prospective applications across diverse fields.

## 5 Conclusion

In recent years, IBD has become a global health challenge with a substantial treatment burden. Research has consistently shown a strong association between the human gut microbiome and IBD pathogenesis, making it crucial for risk prediction. To address the challenges of high-dimensional, sparse, and incomplete microbiome data, this paper introduces a novel integrated data optimization and risk prediction framework, CDORPF. Compared to traditional methods, this approach excels in handling complex microbiome data by preserving the inherent structure of the data, minimizing biases from missing data, and significantly enhancing data integrity and analytical reliability. Additionally, it effectively retains the core information during dimensionality reduction, while markedly improving model predictive performance. This approach offers a comprehensive solution to the challenges of missing values and high dimensionality commonly found in microbiome data.

In clinical workflows, CDORPF can serve as a complementary tool to existing diagnostic methods by providing additional risk assessment information through microbiome analysis. This not only enhances diagnostic accuracy but also optimizes the treatment process, making patient management more refined and personalized. For example, in the initial screening phase, CDORPF can leverage gut microbiome data to help identify high-risk patients, prioritizing further diagnostic or intervention measures and reducing unnecessary delays. Future research can further explore the performance of CDORPF in large-scale, multi-center clinical trials to validate its applicability and robustness across different populations and disease subtypes. Moreover, the successful application of the CDORPF framework offers new research directions for the early diagnosis of other complex diseases, such as cardiovascular disease or cancer, through microbiome analysis, thereby advancing broader applications in personalized medicine.

## Data Availability

Publicly available datasets were analyzed in this study. This data can be found here: https://hmpdacc.org/ihmp/.
